# Endophytic microbiota and metabolites profile in gynoecious versus monoecious cucumbers

**DOI:** 10.1186/s12870-026-09459-w

**Published:** 2026-07-13

**Authors:** Liyuan Liao, Xinyan Zhou, Xinni Li, Weifeng Gu, Kexin Lu, Haidong Huang, Shangdong Yang

**Affiliations:** Guangxi Key Laboratory of Agro-environment and Agro-products Safety, National Demonstration Center for Experimental Plant Science Education, College of Agriculture, Guangxi University, Nanning, 530004 Guangxi P.R. China

**Keywords:** Cucumber (*Cucumis sativus* L.), Sex expression, Microbiome, Metabolome

## Abstract

Sex expression in cucumber (*Cucumis sativus* L.) is a critical agronomic trait governing fruit yield and cultivation efficiency. Although its genetic and hormonal regulation is well-characterized, the role of endophytes and their metabolic interplay remains largely unexplored. In this study, an integrated approach, combining high-throughput sequencing of endophytic bacteria and fungi with untargeted metabolomics was conducted to investigate differences in endophytic community structure between gynoecious versus monoecious cucumbers. We found that gynoecious plants harbored bacterial communities with significantly higher richness, evenness, and a greater number of unique operational taxonomic units (OTUs) than monoecious plants, whereas fungal diversity was not significantly different. Although Proteobacteria, Actinobacteriota, and Firmicutes were dominant in both genotypes, gynoecious were uniquely enriched in Verrucomicrobiota and Myxococcota, while Patescibacteria characterized monoecious. LEfSe analysis identified Myxococcota and Bdellovibrionota as key biomarkers in gynoecious cucumbers, implying a potential for enhancing pathogen suppression. Functional prediction indicated that gynoecious-associated microbiota possessed stronger capacities for hydrocarbon degradation and iron respiration, whereas the microbiota-associated communities were enriched in pathways for nitrogen and nitrate respiration. Metabolomic analyses revealed pronounced genotype-dependent differences, including tryptophan metabolism, plant hormone signal transduction, linoleic and linolenic acid metabolism, and indole alkaloid biosynthesis were significantly upregulated in gynoecious root. Meanwhile, key metabolites, such as L-tryptophan, tryptamine, serotonin, indole-3-acetic acid, jasmonic acid, and salicylic acid were also accumulated at higher levels in gynoecious roots. Furthermore, correlation network analysis revealed stronger associations between specific microbial taxa and hormone- or defense-related metabolites in gynoecious plants compared to monoecious plants.

## Introduction

Cucumber (*Cucumis sativus* L.), an annual vine plant of the family Cucurbitaceae, is indigenous to India and is now cultivated worldwide for its significant economic and nutritional value [1]. Sex expression in cucumber is a key agronomic trait that strongly influences fruit yield and cultivation efficiency. As an important model for studying unisexual flower development [2], cucumber exhibits diverse sexual phenotypes, including monoecious, gynoecious, dioecious, andromonoecious, trimonoecious, and hermaphroditic forms [3]. In agricultural production, gynoecious cucumbers are widely used in protected cultivation and hybrid seed production due to their high proportion of female flowers and strong fruit-setting ability, while monoecious types remain essential in conventional cultivation and parental line development [4].

Sex differentiation in cucumber fundamentally involves the selective suppression of stamens and carpels at specific developmental stages [5]. Early floral buds are bisexual, containing both stamen and carpel primordia. Selective organ arrest during bud development lead to stamen degeneration in female flowers, carpels arrest in male flowers, or no arrest in hermaphroditic flowers [6]. This process exhibits high plastic, influenced by genetic factors, endogenous hormones, and environmental cues. The primary genetic control involves three key loci: *F/f* (dominant *F* strongly promotes femaleness), *M/m* (*M* is required to inhibit stamen development in female flowers), and *A/a* (influencing bisexual flowers formation) [7]. Endogenous hormones mediate these genetic signals, with ethylene and gibberellins acting antagonistically. Ethylene strongly induces femaleness, and expression of its biosynthetic gene *CsACS2*, is regulated in this pathway [8, 9]. Gibberellins, in contrast, primarily promote maleness [10]. Environmental factors, such as short days [11], , low night temperatures [12], and climatic variations [3, 13, 14], further refine this program by altering endogenous hormone balances.

Endophytes are microorganisms that colonize internal plant tissues without causing apparent disease symptoms and are now recognized as integral components of plant holobionts [15]. Increasing evidence demonstrates that endophytic bacteria and fungi play essential roles in plant growth promotion, nutrient acquisition, stress tolerance, disease resistance, and regulation of host metabolism [16–18]. Recent studies have highlighted that endophytic microbiomes can influence plant developmental processes through complex plant–microbe interactions, including hormonal signaling and metabolic reprogramming [19]. Despite growing interest in endophytes, their potential involvement in plant sex differentiation and associated metabolic regulation remains largely unexplored, particularly in economically important crops such as cucumber.

Previous studies had primarily focused on the genetic basis and hormonal regulation of cucumber sex differentiation, but the intrinsic mechanisms underlying the biased development of reproductive organs in different sex types remain incompletely understood. Sex differentiation not only shapes floral morphology and fruit-setting characteristics [20] but also influences plant physiology and metabolism [21–23], indirectly affecting associated endophytic microbial communities [24]. Conversely, the endophytic microbiome can provide feedback, modulating host metabolic homeostasis [25, 26]. Growing evidence shows that plant-associated endophytic bacteria and fungi can produce or modulate multiple phytohormones or their precursors, including indole-3-acetic acid (IAA), cytokinins, ethylene precursors, and gibberellins [27–29], thereby altering host hormone homeostasis and signaling to influence processes such as root growth, tillering, and organ differentiation [30–32]. Metabolomics captures real-time physiological states, bridging genotype, phenotype, and environment [33, 34]. Analyses of floral organs and tissues at different stages have revealed significant differences in amino acids, lipids, phenylpropanoids, and flavonoids between male and female flowers [35]. These metabolites serve as substrates for energy metabolism, cell wall synthesis, and secondary pathways, while also acting as precursors or modulators of hormone biosynthesis to influence organ fate [36]. Additionally, they function as signals regulating microbial adhesion, colonization, and symbiosis [37], forming an important “metabolic fingerprint” for microbiome assembly.

Therefore, this study integrates high-throughput sequencing of endophytic bacteria and fungi with metabolomic profiles in gynoecious and monoecious cucumbers. We systematically characterize the structure, function, and metabolic characteristics of the endophytic microbiome to elucidate how sex differentiation shapes both the endophytic microbial communities and host metabolic networks. This work provides a theoretical foundation for understanding microbial-ecological mechanisms underlying plant sex differentiation and offers novel insights for regulating sex expression and informing breeding strategies in cucumber.

## Materials and methods

### Experimental site and experimental design

The experiment was conducted from March to May 2024 at the Vegetable Teaching and Research Base, College of Agriculture, Guangxi University (108°17′25″E, 22°51′02″N). The site has a subtropical monsoon climate with an annual average temperature of 21.6 °C, a frost-free period of 345–360 days, annual precipitation exceeding 1300 mm, and approximately 1600 h of annual sunshine. The soil is classified as lateritic red earth with the following physicochemical properties: pH 5.64, organic matter 23.26 g·kg⁻¹, total contents of nitrogen, phosphorus and potassium were 1.22 g·kg⁻¹, 0.57 g·kg⁻¹, 6.8 g·kg⁻¹, respectively. Meanwhile, the contents of available nitrogen, phosphorus and potassium were 15.6 mg·kg^− 1^, 0.73 mg·kg^− 1^, 83.3 mg·kg^− 1^, respectively. All cultivars were planted within the same experimental field under identical agronomic management practices to minimize environmental variation among treatments.

**Fig. 1:**
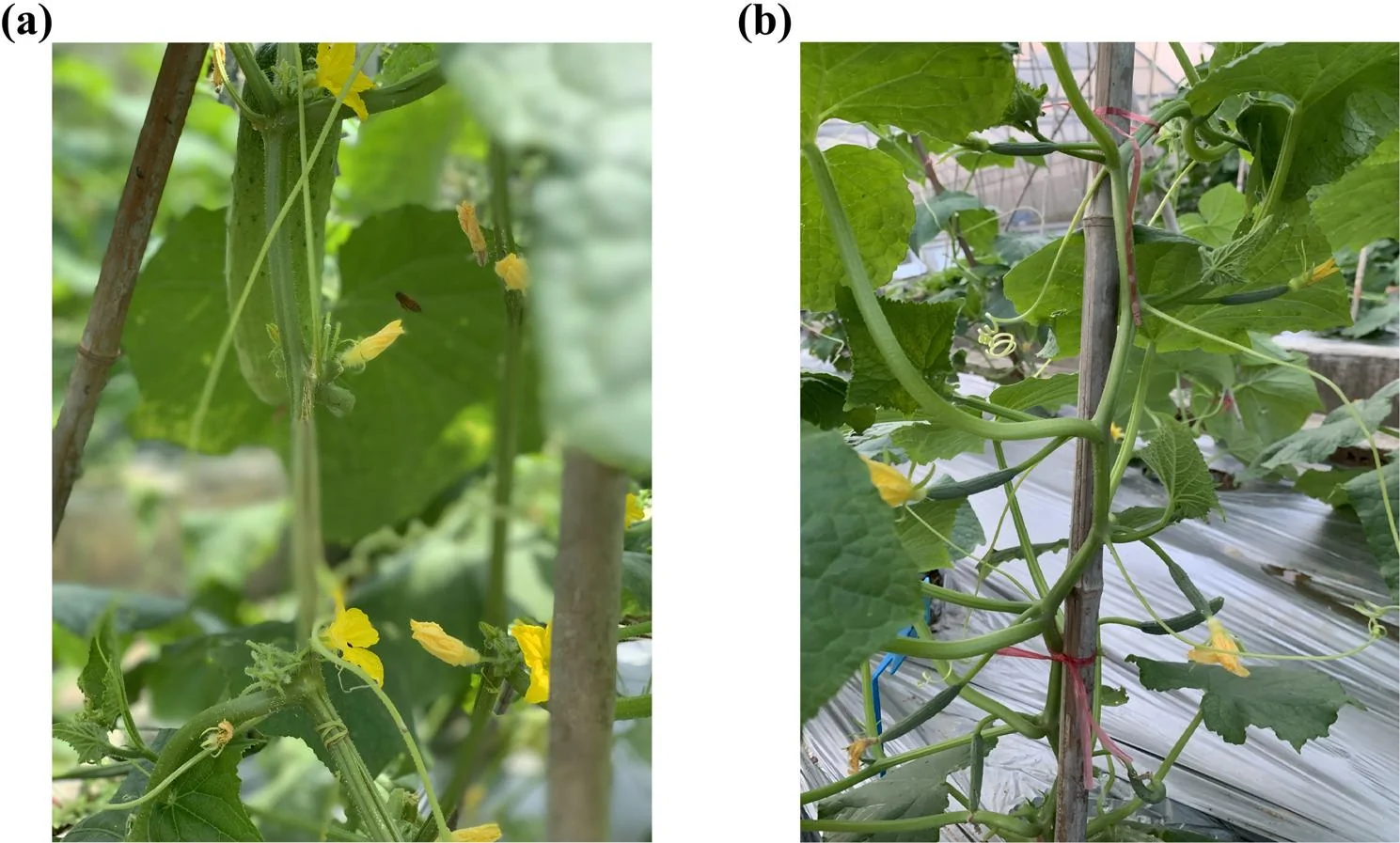
Cucumber cultivation schematic. (**a**) Monoecious; (**b**) Gynoecious

Three gynoecious cucumber cultivars, ‘Ouluoba Yihao’, ‘Meicui 808’, and ‘Shuiguo Huanggua F1’, and three monoecious cultivars, ‘Zhencui 101’, ‘Tiancui’, and ‘Zhenhaochi Jiejiegua’, were used in this study. The three gynoecious cultivars produced only female flowers at each node, with high and stable fruit set rate, smooth and uniform fruits, and good adaptability to protected cultivation. In contrast, the three monoecious cultivars produced male and female flowers sequentially, exhibited vigorous vegetative growth and strong heat tolerance, and set fruits predominantly on the main vine. All experimental materials were F_1_ hybrids, and seed quality conformed to Chinese national standards (purity ≥ 95.0%, cleanliness ≥ 99.0%, germination rate ≥ 85%, moisture content ≤ 8.0%). Seeds were sown in the experimental year, and all plants were cultivated under uniform management conditions. (Fig. 1).

Seeds were germinated in late March 2024, and seedlings were transplanted to the field in April. The experiment followed a randomized complete block design (RCBD) with three replicates per cultivar. All plots received uniform management, including standard irrigation, weeding, and pest control practices.

### Root sample collection and surface sterilization

Roots samples were collected in May 2024. Three healthy and uniformly growing plants per cultivar were randomly selected from each replicate plot. Root samples collected from the three plants within the same replicate plot were pooled to generate one composite biological sample for subsequent microbial community and metabolomic analyses. Thus, each cultivar was represented by three biological replicates, resulting in a total of nine biological samples for each sex-expression type. The roots were carefully excavated, and loosely adhered soil was removed by gently shaking. Fresh root fragments with well-developed lateral roots were excised using sterilized scissors and immediately placed into sterile sealed bags for transport to the laboratory on ice. In the laboratory, root samples were rinsed three times with ultrapure water and gently brushed with a soft brush for 2 min to remove residual debris. Excess water was blotted with sterile filter paper. Root samples were then surface-sterilized by immersion in 2% sodium hypochlorite solution for 10 min, followed by three rinses with sterile ultrapure water, immersion in 70% ethanol for 5 min, and three final rinses with sterile ultrapure water. Surface moisture was removed using sterile absorbent paper.

To verify sterilization efficiency, 100 µL of the final rinse water was spread on Luria-Bertani (LB) agar plates and incubated at 25 °C for 7 days. No microbial colonies growth was observed, confirming effective surface sterilization. Sterilized root samples were flash-frozen in liquid nitrogen and stored at -80℃ until DNA extraction and metabolome analysis.

### DNA extraction, PCR amplification, and sequencing

To comprehensively explore endophytic microbial community structure without prior taxonomic assumptions, high-throughput amplicon sequencing was performed as a non-targeted exploratory analysis. Total genomic DNA was extracted from samples using the E.Z.N.A.^®^ Soil DNA Kit (Omega Bio-tek, Norcross, GA, USA) according to the manufacturer’s instructions. DNA concentration and purity were measured using a NanoDrop 2000 spectrophotometer (Thermo Fisher Scientific, USA). PCR amplification of bacterial 16SrRNA and fungal ITS regions was conducted on an ABI GeneAmp^®^ 9700 thermocycler (Table 1), Amplicons were purified using a DNA Gel Recovery Kit (YuHua, Shanghai, China), quantified with a Qubit 4.0 fluorometer (Thermo Fisher Scientific, USA), and pooled in equimolar amounts. Libraries were conducted using the NEXTFLEX^®^ Rapid DNA-Seq Kit and sequenced on the Illumina MiSeq PE300 platform (Shanghai Majorbio Bio-Pharm Technology Co., Ltd., Shanghai, China). The raw microbial sequencing data have been uploaded to the NCBI SRA (PRJNA1403234 for bacteria; PRJNA1403261 for fungi).

**Table 1:** Primer names and sequences

Primer type	Primer name	Primer sequence (5’-3’)	Sequencing platform	Sequencing length (bp)
bacteria	799 F	AACMGGATTAGATACCCKG	PE300	394
	1193R	ACGTCATCCCCACCTTCC		
fungi	ITS1F	CTTGGTCATTTAGAGGAAGTAA	PE300	242
	ITS2R	GCTGCGTTCTTCATCGATGC		

### Untargeted metabolomic analysis

Untargeted metabolomics was conducted using liquid chromatography–tandem mass spectrometry (LC–MS). Approximately 100 mg of frozen root tissue was homogenized and extracted with 800 µL of pre-chilled methanol–water (4:1, V/V) extraction solvent containing four internal standards, including L-2-chlorophenylalanine (0.02 mg·mL⁻¹), to monitor extraction efficiency and analytical stability. Samples were vortexed, incubated, and centrifuged at 12,000×g for 15 min at 4 °C. Supernatants were transferred to autosampler vials for analysis. Chromatographic separation was performed on a Thermo Fisher Scientific Vanquish UHPLC system equipped with Waters HSS T3C18 column maintained at 40 °C. Mobile phase A was 0.1% formic acid in water, and mobile phase B was 0.1% formic acid in acetonitrile. A gradient elution program was used to ensure effective separation of metabolites. Mass spectrometric data were acquired in both positive and negative ion modes over an m/z range of 50–1200. The raw LC–MS data were processed using Progenesis QI (Waters Corporation, Milford, USA) for baseline filtering, peak detection, integration, retention-time alignment, and peak matching, yielding a data matrix containing retention times, mass-to-charge ratios, and peak intensities for subsequent metabolite identification and multivariate statistical analysis. The processed peak intensity data were normalized using a total signal intensity (sum normalization) method to minimize systematic variation among samples. Features with missing values > 20% were removed, and the remaining missing values were imputed using the minimum value. Variables with relative standard deviation (RSD) > 30% in quality control (QC) samples were excluded. Finally, log10 transformation was applied prior to multivariate statistical analyses. The raw data have been uploaded to the National Genomics Data Center (NGDC, https://ngdc.cncb.ac.cn/omix/), with BioProject accession number PRJCA064591 and OMIX ID OMIX017005.

### Bioinformatic processing and statistical analysis

Preliminary data processing was performed in Microsoft Excel 2019. Microbial community analyses were performed on the I-Sanger cloud platform (Shanghai Majorbio Bio-Pharm Technology Co., Ltd.). Alpha diversity of endophytic microbial communities was assessed using the Simpson index (diversity), Ace index (richness), and evenness Sobs index (evenness). Microbial community composition was analyzed at both the phylum and genus levels. Differential metabolites were identified using partial least squares-discriminant analysis (PLS-DA) with variable importance in projection (VIP) > 1 and *p* < 0.05 (Student’s t-test). Pathway enrichment analysis was performed using the KEGG database.

## Results

### Endophytic bacterial and fungal diversity in gynoecious and monoecious cucumbers

**Fig. 2:**
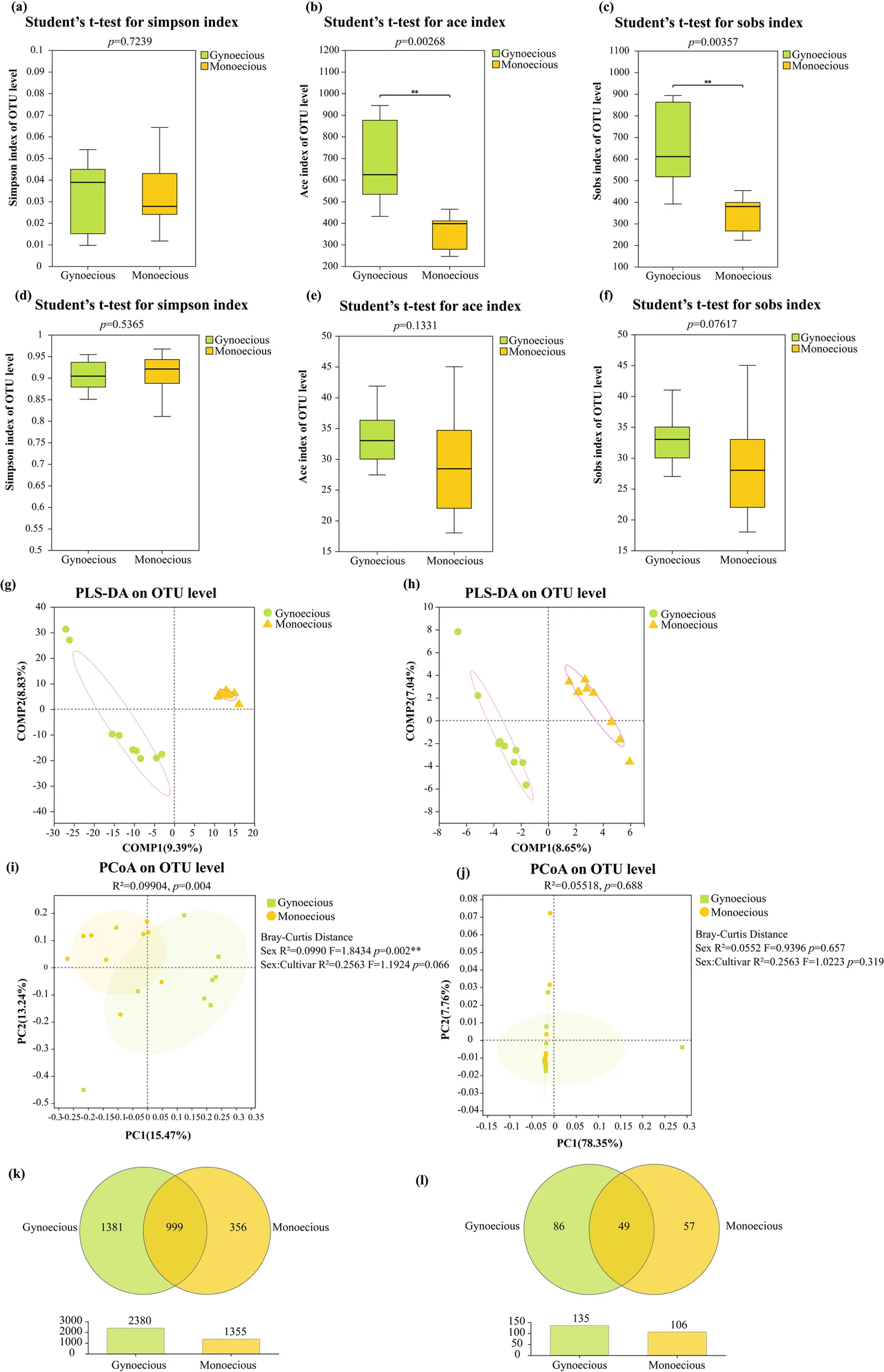
Diversity, PLS-DA and Venn analyses of endophytic microbial communities in gynoecious and monoecious cucumbers. **a** Simpson index of endophyticbacterial communities (diversity); (**b**) Ace index of endophytic bacterial communities (richness); (**c**) Sobs index of endophytic bacterial communities(observed richness); (**d**) Simpson index of endophytic fungal communities (diversity); (e) Ace index of endophytic fungal communities (richness);(f) Sobs index of endophytic fungal communities (observed richness); (**g**) PLS-DA analysis of endophytic bacterial communities; (**h**) PLS-DA analysis ofendophytic fungal communities; (**i**) PCoA analysis of endophytic bacterial communities; (**j**) PCoA analysis of endophytic fungal communities; (**k**) Vennanalysis (OTU level) of endophytic bacterial communities; (**l**) Venn analysis (OTU level) of endophytic fungal communities

As shown in Fig. 2a–c, significant differences were observed in the richness (Ace index and Sobs index) of endophytic bacterial communities between gynoecious and monoecious cucumbers, whereas bacterial diversity (Simpson index) did not differ significantly. In contrast, no significant differences were found in the diversity (Simpson index) or richness (Ace index and Sobs index) of endophytic fungal communities between the two cucumber types (Fig. 2d–f).

PLS-DA (Partial Least Squares Discriminant Analysis) was performed to evaluate the similarity of endophytic microbial communities between gynoecious and monoecious cucumbers. The endophytic bacterial communities of the two cucumber types formed clearly separated clusters (Fig. 2g), indicating distinct compositional and structural differences. Similarly, the endophytic fungal communities in roots also showed a clear separation consistent with the bacterial clustering pattern (Fig. 2h). PCoA based on Bray–Curtis distance revealed that the bacterial communities were significantly different between gynoecious and monoecious cucumbers (R²=0.09904, *p* = 0.004; Fig. 2i), while no significant difference was observed for fungal communities (R²=0.05518, *p* = 0.688; Fig. 2j). These findings are further supported by nested PERMANOVA analyses, which confirmed that the significant community separation observed in bacteria remained robust after accounting for cultivar effects, whereas the apparent separation in fungi was not statistically significant when cultivar background was controlled.

Venn diagram analysis at the OTU level revealed that gynoecious cucumbers contained 1,381 unique endophytic bacterial OTUs, substantially more than the 356 OTUs unique to monoecious cucumbers (Fig. 2k). Similarly, the number of unique endophytic fungal OTUs was higher in gynoecious roots (86) than in monoecious roots (Fig. 2l). These results indicate that the gynoecious genotype harbors a more diverse and abundant set of unique endophytic microorganisms than the monoecious genotype, suggesting genotype-specific recruitment of endophytic microbial communities in cucumber roots.

### Endophytic bacterial and fungal community compositions in gynoecious and monoecious cucumbers

**Fig. 3:**
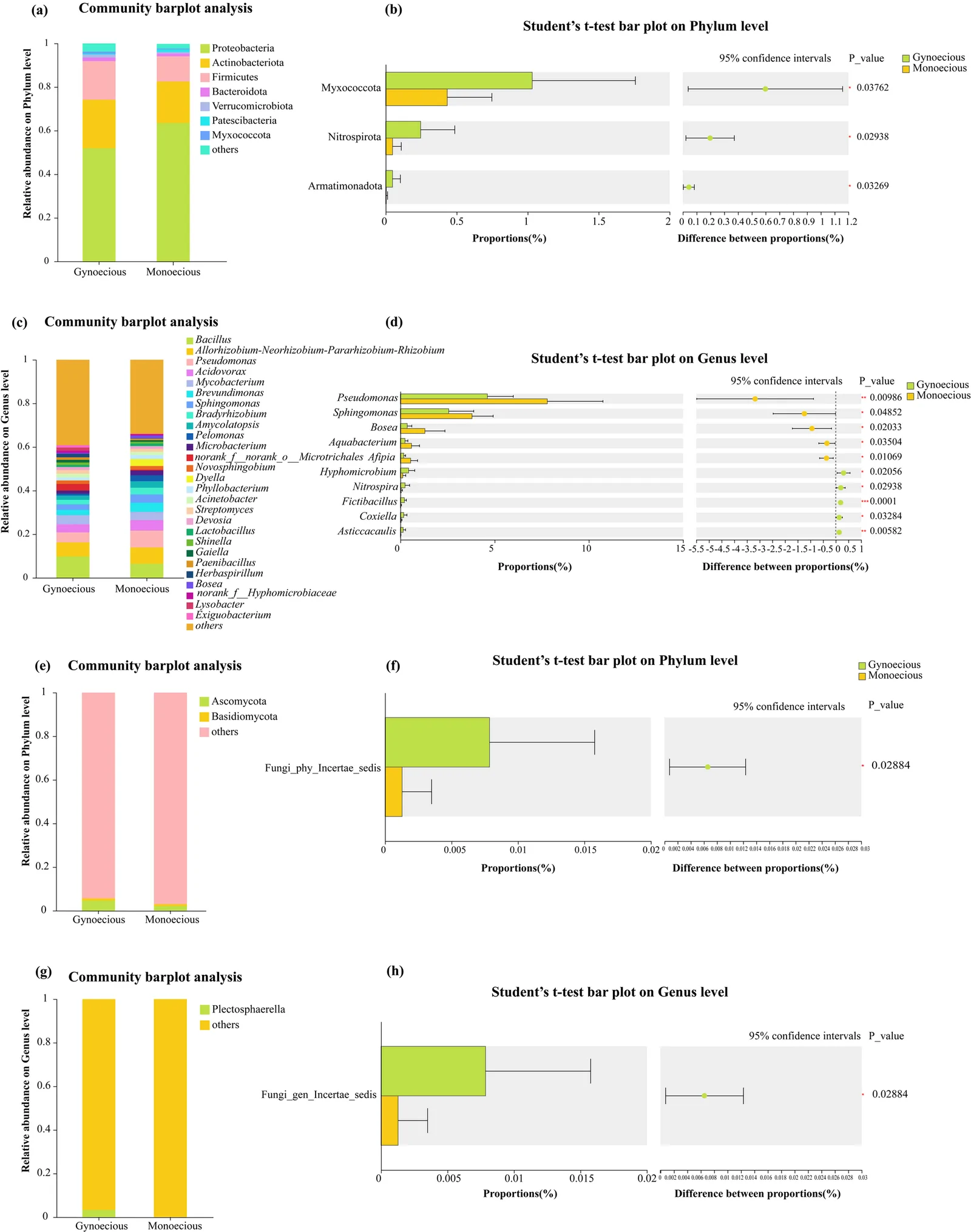
Composition of endophytic microbial communities in gynoecious and monoecious cucumber roots at the phylum and genus levels. **a **Relativeabundance of bacterial communities at the phylum level; (**b**) Significant differences in bacterial phyla between the two cucumber types; (**c**) Relativeabundance of bacterial communities at the genus level; (**d**) Significant differences in bacterial genera between the two cucumber types; (**e**) Relativeabundance of fungal communities at the phylum level; (**f**) Significant differences in fungal phyla between the two cucumber types; (**g**) Relative abundanceof fungal communities at the genus level; (**h**) Significant differences in fungal genera between the two cucumber types

At the phylum level, six dominant bacterial phyla (relative abundance > 1%) were detected in gynoecious cucumber roots, compared with five in monoecious cucumbers. The three most abundant phyla in both genotypes were Proteobacteria, Actinobacteriota, and Firmicutes, which showed similar relative abundances. Verrucomicrobiota and Myxococcota were uniquely dominant phyla in gynoecious roots, whereas Patescibacteria was unique to monoecious roots (Fig. 3a). At the genus level, 25 and 19 dominant bacterial genera were identified in gynoecious and monoecious roots, respectively. Genera including Norank_f__norank_o__*Microtrichales*, *Shinella*, *Gaiella*, *Paenibacillus*, *Herbaspirillum*, norank_f__*Hyphomicrobiaceae*, *Lysobacter*, and *Exiguobacterium* were specific to gynoecious cucumbers, while *Pelomonas*, *Dyella*, *Lactobacillus*, *Bosea* were specific to monoecious cucumbers (Fig. 3c). Wilcoxon rank-sum tests confirmed significant differences in the abundance of three bacterial phyla (Fig. 3b) and ten bacterial genera (Fig. 3d) between the two genotypes.

For fungal communities, two dominant phyla were detected in gynoecious roots, whereas only one was observed in monoecious roots. Basidiomycota was uniquely present in gynoecious roots, with no unique dominant fungal phyla were detected in monoecious roots (Fig. 3e). At the genus level, only one dominant fungal genus, *Plectosphaerella*, was detected in gynoecious roots, while none were identified in monoecious roots (Fig. 3g). Wilcoxon rank-sum tests indicated significant differences in the abundance of only one fungal phylum (Fig. 3f) and one fungal genus (Fig. 3h).

**Fig. 4:**
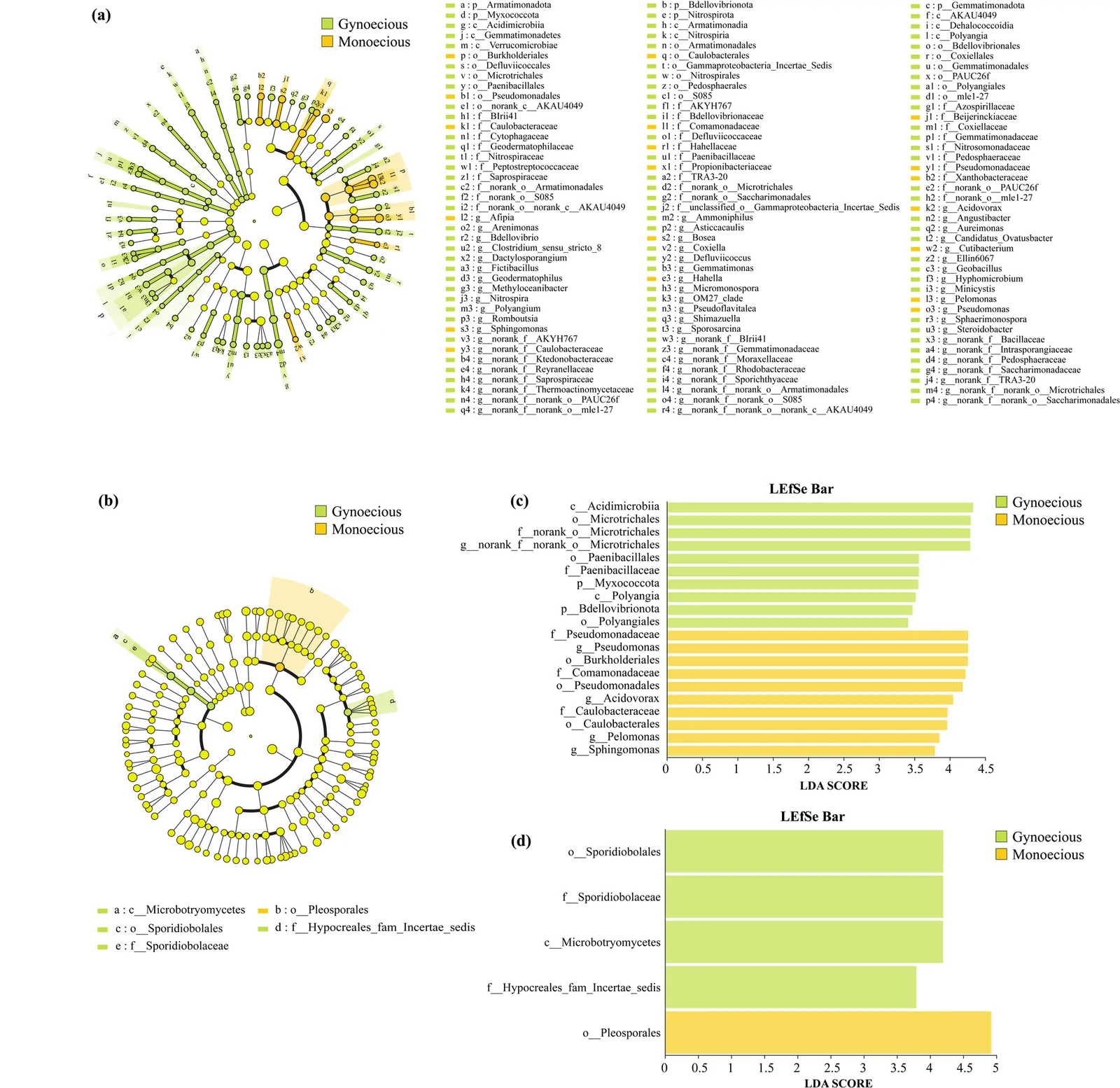
LEfSe analysis of endophytic bacterial and fungal communities (LDA 3.0). **a** Multilevel taxonomic cladogram of bacterial communities; **b** Multileveltaxonomic cladogram of fungal communities; (**c**) LDA score bar plot of bacterial communities; (**d**) LDA score bar plot of fungal communities

Linear discriminant analysis effect size (LEfSe) analysis (LDA threshold > 3.0) was used to identify differentially abundant taxa and key biomarkers between the endophytic bacterial and fungal communities of the two cucumber types. At the phylum level, Myxococcota and Bdellovibrionota were significantly enriched in gynoecious roots. At the genus level, norank_f__norank_o__*Microtrichales* was significantly enriched in gynoecious roots, whereas *Pseudomonas*, *Acidovorax*, *Pelomonas*, and *Sphingomonas* were significantly enriched in monoecious roots (Figs. 4a, c). In contrast, no fungal phyla or genera showed significant enrichment in either gynoecious or monoecious roots (Figs. 4b, d).

### Functional prediction of endophytic microbial communities

**Fig. 5:**
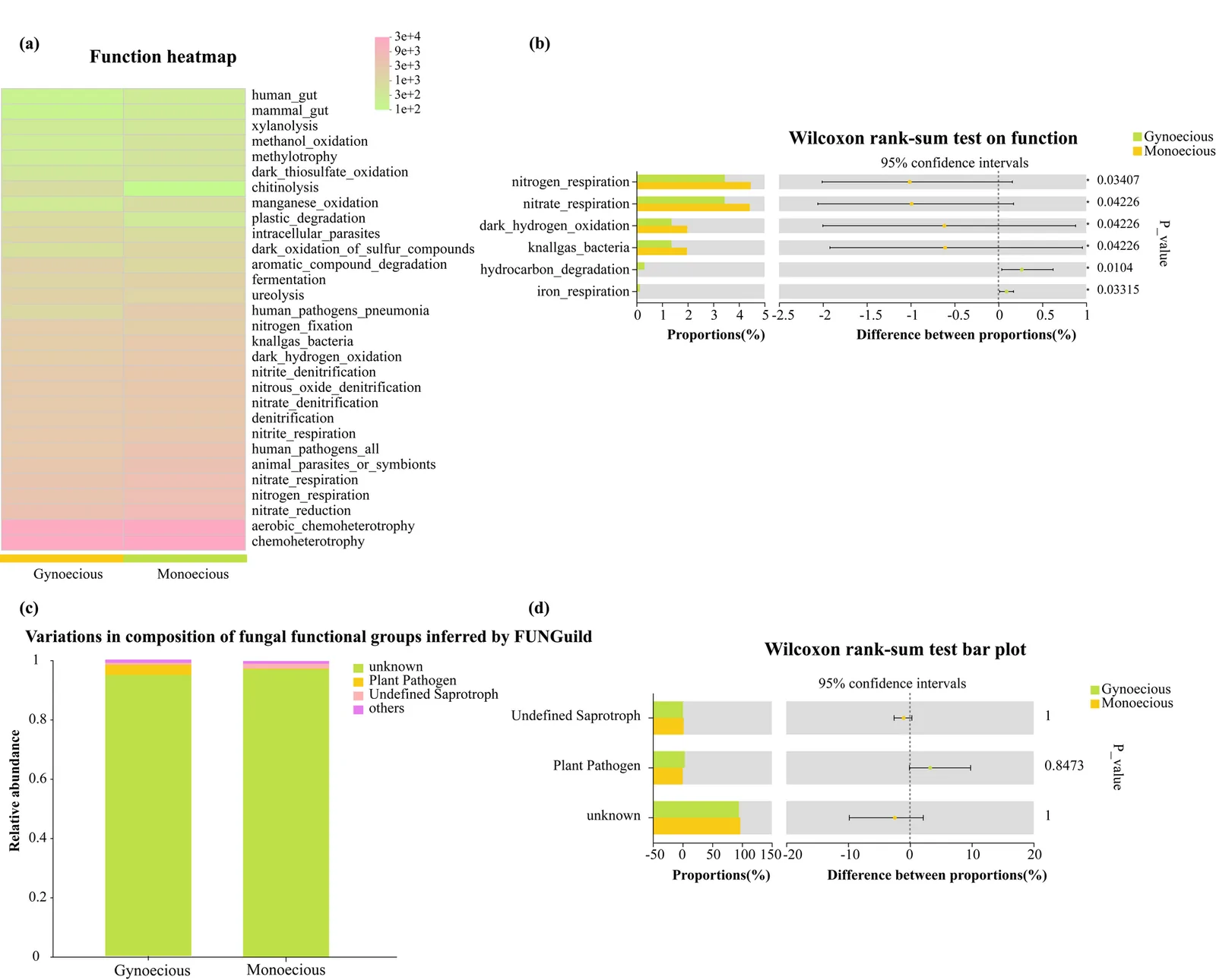
Functional prediction of endophytic microbial communities in gynoecious and monoecious cucumbers. **a** Bacterial functional potential inferredusing FAPROTAX. **b** Bacterial functions predicted to differ in relative abundance between genotypes (Wilcoxon rank-sum test). **c** Fungal trophic modeassigned by FUNGuild. **d** Fungal functional guilds predicted to differ in relative abundance between genotypes (Wilcoxon rank-sum test)

Functional predictions of bacterial communities were generated using the FAPROTAX database, revealing distinct predicted functional profiles between gynoecious and monoecious cucumbers (Fig. 5a). Wilcoxon rank-sum tests identified several functions predicted to differ between the two genotypes (Fig. 5b). Predicted hydrocarbon degradation and iron respiration functions were more abundant in gynoecious cucumbers, whereas predicted nitrogen respiration, nitrate respiration, dark hydrogen oxidation, and knallgas bacteria–associated functions were more abundant in monoecious cucumbers. For fungal communities, functional guild composition was inferred using FUNGuild (Fig. 5c). Notably, most endophytic fungal taxa could not be assigned to specific guilds, and undefined saprotrophs dominated the dataset. Limited by the low resolution of ITS-based taxonomy and guild annotation, no significant differences in predicted trophic modes or functional guilds were detected between the two cucumber types (Fig. 5d).

### Analysis of root metabolites in gynoecious and monoecious cucumbers

The root metabolite profiles of gynoecious and monoecious cucumbers were analyzed using untargeted (LC–MS) metabolomics (Table 2). The analysis yielded a total of 18,469 metabolite ion peaks and detected 873 metabolites. Subsequently, 770 metabolites were annotated against public databases, among which 477 were assigned identities in the KEGG database.

**Table 2:** Total ion counts and metabolite identification statistics in gynoecious and monoecious cucumber roots

Ion mode	All peaks	Identified metabolites	Metabolites in library	Metabolites in KEGG
pos	10,898	530	457	293
neg	7571	343	313	184
Total	18,469	873	770	477

**Fig. 6:**
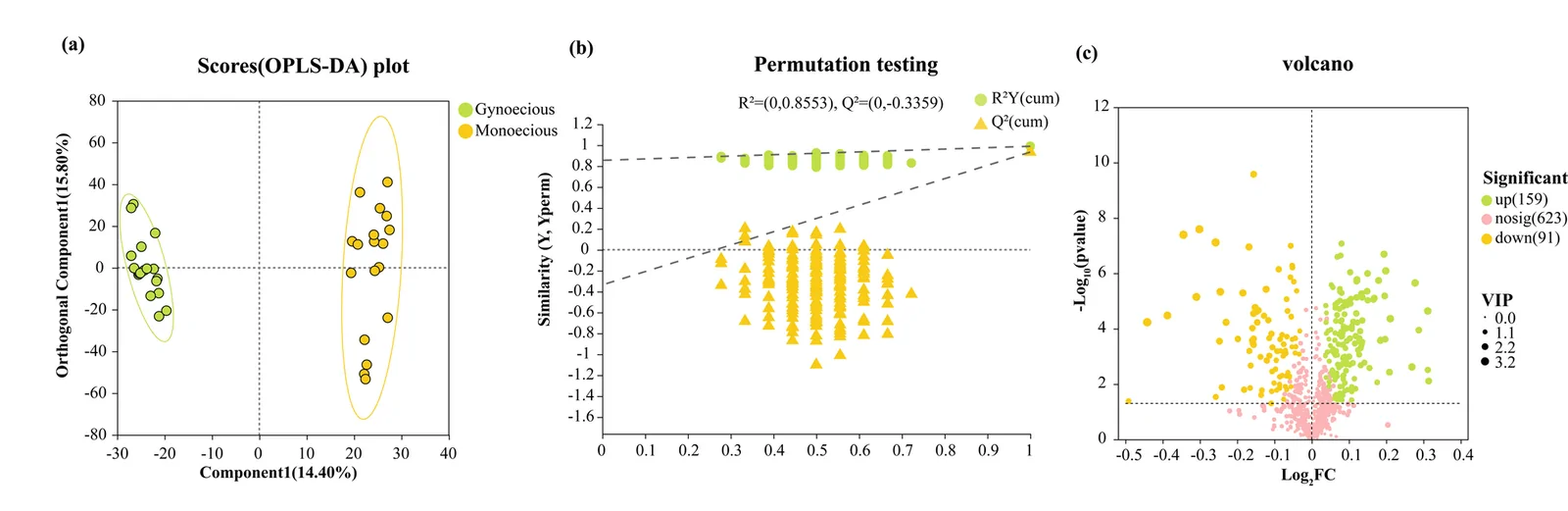
Comparison of metabolites between gynoecious and monoecious cucumbers. **a** OPLS–DA score plot of root metabolites.** b** Permutation test ofthe OPLS–DA model. **c** Volcanic plot of differential metabolites

OPLS–DA analysis of root metabolites from gynoecious and monoecious cucumbers (mix mode) showed that the first predictive component explained 14.4% of the variance, while the first orthogonal component accounted for 15.8%. Samples s from each genotype exhibited strong within-group clustering and reproducibility, forming clearly separated clusters, which indicated significant differences in root metabolite composition between the two genotypes (Fig. 6a). To validate the model, 200 permutation tests were performed. Model fit and predictive ability were evaluated using R²Y and Q² values; higher values reflect greater model stability and reliability. In the mix mode, the cumulative R²Y and Q² values reached 0.992 and 0.939, respectively (Fig. 6b). Throughout the permutation tests, the randomly generated R² and Q² values remained substantially lower than the original values. Moreover, all regression line intercepts for Q² were below zero, confirming that the model was not overfitted. Variable importance in projection (VIP) values were subsequently used to select relevant metabolites. Different metabolites between gynoecious and monoecious roots were identified by applying thresholds of VIP > 1 and *p* < 0.05. Based on these criteria, 250 differential metabolites were detected, with 159 upregulated and 91 downregulated in gynoecious roots compared to monoecious roots (Fig. 6c).

**Fig. 7:**
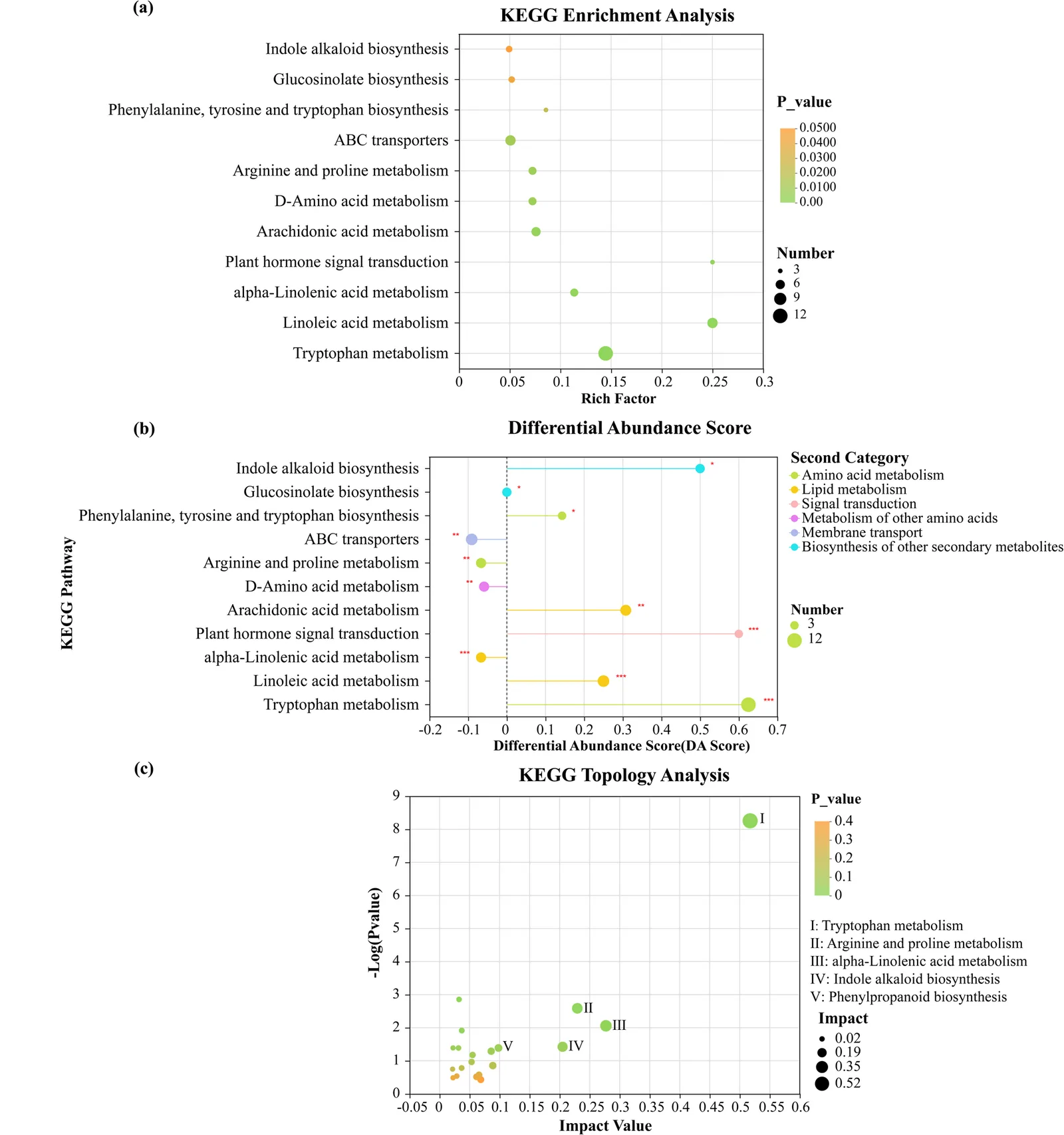
KEGG pathway enrichment analysis of differential metabolites in gynoecious and monoecious cucumber roots. **a** Enrichment bubble plot of KEGGpathways; (**b**) Differential abundance (DA) score plot; (**c**) Bubble plot of pathway topology analysis showing impact factors

The 250 differential metabolites were annotated against the KEGG PATHWAY database. Pathway enrichment analysis was performed using a hypergeometric distribution algorithm to identify significantly altered metabolic pathways. The results revealed 11 root metabolic pathways that were significantly different between gynoecious and monoecious cucumbers (Fig. 7a). Among these pathways, the gynoecious genotype exhibited significantly higher activity in tryptophan metabolism, linoleic acid metabolism, plant hormone signal transduction, arachidonic acid metabolism, phenylalanine, tyrosine and tryptophan biosynthesis, and indole alkaloid biosynthesis compared with monoecious cucumbers. In contrast, α-linolenic acid metabolism, D-amino acid metabolism, arginine and proline metabolism, and ABC transporters were significantly lower in gynoecious roots. (Fig. 7b). Furthermore, KEGG topology analysis was performed to assess pathway impact. The five pathways with the highest impact scores were tryptophan metabolism, arachidonic acid metabolism, α-linolenic acid metabolism, indole alkaloid biosynthesis, and phenylalanine, tyrosine and tryptophan biosynthesis (Fig. 7c).

Of the 61 differential metabolites annotated to KEGG metabolic pathways, 39 were significantly more abundant in gynoecious roots, while 22 were significantly less abundant compared with monoecious roots (Table 3).

**Table 3:** Significantly different metabolites between gynoecious and monoecious cucumbers

Differential metabolic pathways	Metabolite	Regulate	Formula	Mode
Phenylalanine, tyrosine and tryptophan biosynthesis	L-Tryptophan	up	C11H12N2O2	pos
	3-Hydroxybenzoic Acid	up	C7H6O3	neg
	Phenylpyruvic Acid	down	C9H8O3	neg
Plant hormone signal transduction	Salicylic Acid	up	C7H6O3	pos
	Jasmonic acid	up	C12H18O3	pos
	Indole-3-acetic acid	up	C10H9NO2	pos
Glucosinolate biosynthesis	L-Tryptophan	up	C11H12N2O2	pos
	Glucoerucin	up	C12H23NO9S3	pos
	L-isoleucine	down	C6H13NO2	pos
	3-Methyl-2-Oxovaleric Acid	down	C6H10O3	neg
Indole alkaloid biosynthesis	L-Tryptophan	up	C11H12N2O2	pos
	Harman	up	C12H10N2	pos
	Secologanin	down	C17H24O10	neg
	Tryptamine	up	C10H12N2	neg
D-Amino acid metabolism	D-glutamine	up	C5H10N2O3	pos
	L-proline	down	C5H9NO2	pos
	Putrescine	down	C4H12N2	pos
	Phenylpyruvic Acid	down	C9H8O3	neg
	5-Aminovaleric Acid	up	C5H11NO2	neg
Arginine and proline metabolism	4-Guanidinobutanoic Acid	up	C5H11N3O2	pos
	L-proline	down	C5H9NO2	pos
	Putrescine	down	C4H12N2	pos
	5-Aminovaleric Acid	up	C5H11NO2	neg
	N2-Succinyl-L-ornithine	down	C9H16N2O5	neg
alpha-Linolenic acid metabolism	Jasmonic acid	up	C12H18O3	pos
	12-opda	down	C18H28O3	pos
	13-hotre	down	C18H30O3	pos
	13(S)-Hydroperoxylinolenic acid	up	C18H30O4	neg
	Traumatin	down	C12H20O3	neg
Arachidonic acid metabolism	20-Hydroxy-leukotriene B4	up	C20H32O5	pos
	Prostaglandin J2	up	C20H30O4	pos
	11 h-14,15-eeta	up	C20H32O4	pos
	Prostaglandin-c2	up	C20H30O4	pos
	14,15-dhet	up	C20H34O4	pos
	20-Hydroxyeicosatetraenoic acid	down	C20H32O3	pos
ABC transporters	D-sorbitol	down	C6H14O6	pos
	L-isoleucine	down	C6H13NO2	pos
	Deoxyadenosine	up	C10H13N5O3	pos
	L-proline	down	C5H9NO2	pos
	Putrescine	down	C4H12N2	pos
	Phosphate	down	H3O4P	neg
	Phthalic Acid	up	C8H6O4	neg
Linoleic acid metabolism	9-OxoODE	up	C18H30O3	pos
	9,12,13-TriHOME	up	C18H34O5	neg
	9(S)-HpODE	up	C18H32O4	neg
	9,10-dhome	up	C18H34O4	neg
	Dihomo-gamma-linolenic acid	up	C20H34O2	neg
	13(S)-HpODE	down	C18H32O4	neg
	12,13-EpOME	down	C18H32O3	neg
Tryptophan metabolism	5-Hydroxy-L-tryptophan	up	C11H12N2O3	pos
	Serotonin	up	C10H12N2O	pos
	L-Tryptophan	up	C11H12N2O2	pos
	5-Methoxyindole-3-Acetic Acid	up	C11H11NO3	pos
	Indole-3-acetonitrile	up	C10H8N2	pos
	6-hydroxymelatonin	up	C13H16N2O3	pos
	Tryptophol	up	C10H11NO	pos
	Indole-3-acetic acid	up	C10H9NO2	pos
	3-(3-indolyl)-2-oxopropanoic acid	up	C11H9NO3	pos
	L-Formylkynurenine	up	C11H12N2O4	pos
	Picolinic Acid	down	C6H5NO2	pos
Tryptophan metabolism	Tryptamine	up	C10H12N2	neg

### Correlation analysis between microorganisms and metabolites

**Fig. 8:**
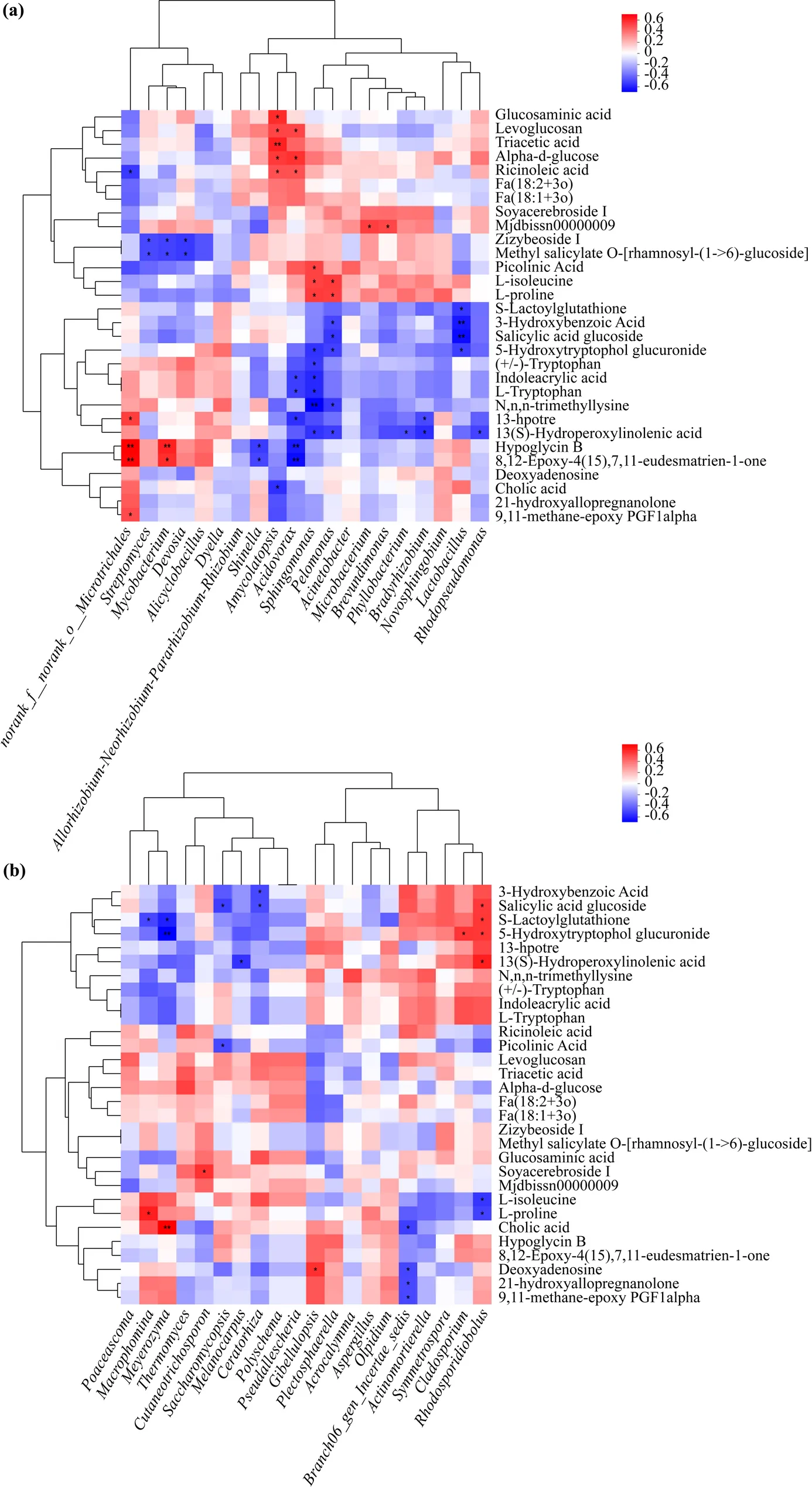
Correlations between metabolites and endophytic microbial communities in gynoecious and monoecious cucumbers. **a** Heatmap showing correlationsof the top 30 metabolites with bacterial genera. **b** Heatmap showing correlations of the top 30 metabolites with fungal genera. * 0.01 <* p* < 0.05,** 0.001 < *p* < 0.01, *** *p* < 0.001

Spearman correlation analysis was conducted to assess associations between root metabolites and endophytic microbial genera. Several bacterial genera, including *Bosea*, *Amycolatopsis*, and *Acidovorax*, showed significant positive associations with carbohydrate metabolites (e.g., Glucosaminic acid, Levoglucosan, Triacetic acid, and α-D-glucose) and fatty acid metabolites (e.g., Ricinoleic acid, FA (18:2 + 3O), FA (18:1 + 3O)). Conversely, *Bosea* and *Acidovorax* were negatively associated with oxylipins and glutathione-related metabolites, such as S-Lactoylglutathione, 13-HPOTre, and epoxy-prostaglandins. Genera such as *Pseudomonas*, *Sphingomonas*, and *Pelomonas* were broadly positively associated with amino acid metabolites (Picolinic acid, L-isoleucine, and L-proline) but negatively associated with N, N, N-trimethyllysine and various indole-related metabolites, including L-tryptophan, Indoleacrylic acid, and 5-Hydroxytryptophol glucuronide. Several plant defense–related oxylipins, including 13(S)-Hydroperoxylinolenic acid and 13-HPOTre, were significantly negatively associated with *Phyllobacterium*, *Bradyrhizobium*, and *Melanocarpus*, whereas they were positively associated with *Gaiella*, *Paenibacillus*, and norank_f__*Hyphomicrobiaceae*. Additionally, *Streptomyces*, *Devosia*, and *Mycobacterium* were significantly negatively associated with flavonoid glycosides (e.g., Zizybeoside I) and salicylate glycoside–type defense compounds. In contrast, genera such as norank_f__*Microtrichales*, *Paenibacillus*, *Gaiella*, and *Hyphomicrobiaceae* displayed significant positive associations with toxic amino acid derivatives and sesquiterpenoids (e.g., Hypoglycin B and 8,12-epoxy eudesmatrien-1-one) (Fig. 8a). Among fungal genera, *Macrophomina*, *Cutaneotrichosporon*, and *Cladosporium* were positively associated with L-proline, Soyacerebroside I, or 5-Hydroxytryptophol glucuronide, respectively. In contrast, genera such as *Meyerozyma*, *Saccharomycopsis*, *Ceratorhiza*, and *Rhodosporidiobolus* were primarily negatively associated with salicylic acid derivatives, benzoic acid–related metabolites, and glutathione-associated metabolites (Fig. 8b).

## Discussions

Sex differentiation in dioecious plants is determined not only by genetic factors-such as genic balance modification, sex chromosomes, and sex-determining genes [38, 39], but also by phytohormonal regulation and multiple environmental factors, including temperature, photoperiod, and nutrient availability [40–42]. Recent advances in plant–microbe interaction research have highlighted the plant microbiome as a critical external regulator of host phenotypes, playing key roles in plant growth, development, stress adaptation, and metabolic regulation [43–46]. Consequently, plants may obtain adaptive advantages by regulating the assembly of their associated microbial communities, a process with important evolutionary ecological implications.

The root endophytic bacterial communities of gynoecious cucumbers exhibited significantly higher richness and evenness than those of monoecious plants and harbored substantially more unique OTUs. This suggests that the root environment of gynoecious plants may offer more diverse and selective ecological niches, thereby favoring the colonization of specific functional microorganisms and fostering a distinct endophytic microbial reservoir. Although overall fungal community diversity did not differ significantly, gynoecious plants also hosted a greater number of unique fungal OTUs. Community composition analysis showed that Proteobacteria, Actinobacteriota, and Firmicutes dominated both cucumber types, consistent with previous studies [47, 48]. Notably, Verrucomicrobiota and Myxococcota were uniquely enriched in gynoecious plants; these taxa are often linked to carbon utilization, predatory behavior, and enhanced plant defense. Members of Verrucomicrobiota are genetically equipped for polysaccharide degradation, enabling them to use complex plant-derived carbon sources and participate in soil carbon cycling [49, 50]. Myxococcota, known for their predatory and antagonistic activities, may indirectly enhance plant defense by suppressing soil-borne pathogens or modulating root endophytic community structure [51, 52]. In contrast, monoecious cucumbers were enriched in Patescibacteria (CPR bacteria), which possess ultra-small cells, highly reduced genomes, limited metabolic capacity, and strong dependence on hosts or other microorganisms for nutrients [53, 54], indicative of a more “resource-dependent” root endophytic ecosystem.

At the genus level, gynoecious cucumbers were enriched in typical functional taxa such as *Paenibacillus* and *Shinella*, which are associated with phytohormone (e.g., IAA) biosynthesis, stress-related metabolism, nitrogen cycling, and organic matter degradation [55, 56]. Their enrichment may contribute to optimized nutrient acquisition and overall plant adaptability. Monoecious plants, conversely, were enriched in genera including *Pelomonas*, *Dyella*, *Bosea*, and *Sphingomonas*, some of which participate in nitrogen respiration, lipid degradation, or oxidative stress metabolism [57–61]. These patterns suggest that genotypic differences may shape microbial colonization preferences through shifts in the metabolite profiles. LEfSe analysis further supported this biased distribution: enrichment of Myxococcota and Bdellovibrionota in gynoecious plants points to greater potential for pathogen suppression [62, 63], whereas enrichment of *Pseudomonas* and *Sphingomonas* in monoecious plants reflects typical functional bacteria that may favor nutrient assimilation and enhance basal metabolism [64, 65].

Functional prediction of microbial communities indicated that the root microbiota of gynoecious plants possessed stronger potential for hydrocarbon degradation and iron respiration, whereas microbial communities in monoecious plants were more prominent in nitrogen respiration, nitrate respiration, and hydrogen oxidation. These functional niche differences aligned closely with root metabolite profiles, suggesting that the root metabolic environment drives functional restructuring of the associated microbiota.

Compared with monoecious plants, multiple key metabolic pathways were significantly enriched in gynoecious roots, including tryptophan metabolism, plant hormone signal transduction, linoleic and linolenic acid metabolism, aromatic amino acid biosynthesis, and indole alkaloid metabolism. Notably, key metabolites in the tryptophan pathway—such as L-tryptophan, tryptamine, serotonin, and their downstream product indole-3-acetic acid (IAA)—accumulated markedly in gynoecious roots. Tryptophan is the principal precursor of IAA [66], which is e a critical hormone regulating female flower differentiation [67]; elevated endogenous or exogenous IAA levels can significantly increase the proportion of female flowers [68]. Thus, enhanced tryptophan metabolism in gynoecious roots may influence aboveground sex expression via root-to-shoot hormone transport. Additionally, jasmonic acid (JA) and salicylic acid (SA) were also significantly upregulated in gynoecious roots. JA is known to play a central role in stamen development, anther dehiscence, and floral organ maturation [69], and extensive crosstalk among JA, auxin, gibberellin, and ethylene signaling pathways jointly determine floral organ fate [70]. SA can induce stamen degeneration in many monoecious or dioecious plants; in Vernicia fordii, SA accumulation is closely associated with programmed cell death (PCD) of stamens and represents a key signal leading to male organ degeneration and female flower formation [71]. Although the biological significance of the differential accumulation of JA, SA, and their derivatives in cucumber roots remains unclear, these results suggest potential links between root metabolic status and reproductive development and warrant further investigation. Oxylipin metabolism also exhibited pronounced differences: increased in 13(S)-HpODE and the decreased leves of certain oxylipins in gynoecious plants suggest that root-derived oxidative lipid signals may be involved in microbial selection and root immune regulation.

Collectively, our findings suggest that gynoecious cucumbers establish a functionally diverse root microecosystem enriched in mutualistic microorganisms by strengthening the tryptophan–IAA pathway, activating JA and SA signaling, and promoting the accumulation of specific secondary metabolites. In contrast, monoecious cucumbers, characterized by nitrogen-metabolizing microbial dominance and weaker hormone-related metabolism, may exhibit a distinct root ecological strategy. These results indicate that cucumber sex-expression type may play an important role in shaping root-associated microbial communities and metabolic profiles. However, because the gynoecious and monoecious materials used in this study represent different commercial cultivars rather than near-isogenic lines, the potential influence of cultivar-specific genetic background cannot be completely excluded. Therefore, the observed microbial and metabolic differences should be interpreted as being associated with sex-expression type, while further studies using genetically controlled materials are needed to verify the extent to which sex expression itself contributes to the assembly of root microbial communities and metabolic characteristics.

## Conclusions

Gynoecious cucumbers possess root endophytic bacteria with higher diversity and distinct composition relative to monoecious cucumbers. Functional and antagonistic genera including *Paenibacillus*, *Shinella*, Myxococcota and Bdellovibrionota are highly enriched in gynoecious roots, while monoecious roots are dominated by bacteria with limited metabolic capacities and strong resource dependence. Metabolically, gynoecious roots show enhanced activity in the tryptophan-IAA pathway, plant hormone signaling, jasmonic acid, salicylic acid and oxylipin metabolism. By contrast, monoecious roots are mainly characterized by nitrogen metabolism, with low activity in hormone and secondary metabolite pathways. These coordinated differences in microbial composition and metabolic function likely establish an internal root environment that is more conducive to female flower differentiation and reproductive development in gynoecious cucumbers. Collectively, our findings demonstrate that the structure and functional potential of root endophytic microbial communities are closely linked to cucumber sex type and can be systematically reshaped by the root metabolic environment. Thus, sex differentiation is not solely determined by genetic and hormonal regulation but is also intimately influenced by root metabolism–microbiome interactions.

## Data Availability

The datasets generated and analysed during the current study are available in public repositories as follows: Raw bacterial and fungal microbial sequencing data were deposited in the NCBI Sequence Read Archive (SRA, https://www.ncbi.nlm.nih.gov/sra), with BioProject accession numbers PRJNA1403234 (bacteria) and PRJNA1403261 (fungi). Untargeted metabolomics raw datasets were submitted to the National Genomics Data Center (NGDC, https://ngdc.cncb.ac.cn/omix/), with BioProject accession PRJCA064591 and OMIX identifier OMIX017005.
